# On the OCRA Measurement: Automatic Computation of the Dynamic Technical Action Frequency Factor

**DOI:** 10.3390/s20061643

**Published:** 2020-03-16

**Authors:** Juri Taborri, Marco Bordignon, Francesco Marcolin, Alessandro Bertoz, Marco Donati, Stefano Rossi

**Affiliations:** 1 Department of Economics, Engineering, Society and Business Organization (DEIM), University of Tuscia, 01100 Viterbo, VT, Italy; stefano.rossi@unitus.it; 2 ErgoCert—Ente di Certificazione per l’Ergonomia srl, 33100 Udine, UD, Italy; marco.bordignon@ergocert.it (M.B.); francesco.marcolin@ergocert.it (F.M.); alessandro.bertoz@ergocert.it (A.B.); 3 Motustech—Sport & Health Technology c/o Marilab, 00121 Roma, RM, Italy; marco.donati@motustech.it

**Keywords:** OCRA, technical actions, upper limb musculoskeletal disorders, threshold-based algorithm, inertial sensors

## Abstract

OCRA (OCcupational Repetitive Action) is currently one of the most widespread procedures for assessing biomechanical risks related to upper limb repetitive movements. Frequency factor of the technical actions represents one of the OCRA elements. Actually, the frequency factor computation is based on workcycle video analysis, which is time-consuming and may lead to up to 30% of intra-operator variability. This paper aims at proposing an innovative procedure for the automatic counting of dynamic technical actions on the basis of inertial data. More specifically, a threshold-based algorithm was tested in four industrial case studies, involving a cohort of 20 workers. Nine combinations of the algorithm were tested by varying threshold values related to time and amplitude. The computation of frequency factor showed an average relative error lower than 5.7% in all industrial-based case studies after the appropriate selection of the time and amplitude threshold values. These findings open the possibility to use the threshold-based algorithm proposed here for the automatic computation of OCRA frequency factor, avoiding the time efforts in video analysis.

## 1. Introduction

Excessive workloads represent one of the main factors known to be potentially dangerous for the occurrence of Musculoskeletal Disorders (MSDs) [1]. According to an American survey, more than 30% of occupational injuries are due to MSD; among them, 2% are caused by the repetitive movements during the work cycle [2]. The frequency of movements, exerted manual forces and awkward postures have been demonstrated to be the main risk factors for MSDs related to the upper limbs, regardless complementary physical, psychological and environment factors [3,4].

Through the aim to quantify the risk of MSDs, several semi-quantitative and observational risk assessment tools are currently available, such as Strain Index, TLV (Threshold Limit Values) and OCRA (OCcupational Repetitive Action) [1]. Several studies demonstrated as the outcomes of the above-mentioned assessment tools are often in disagreement with each other since the examined variables and the used computational algorithms are different [5,6].

Focusing on OCRA index, it is widely recognized as the most complete method for an analytical evaluation and it is also the most widespread for assessing biomechanical risk related to repetitive tasks performed by upper limbs [7]. In addition, the ISO 11228-3 suggests using this index as “preferred” one due to the provided link with the expected percentage of MSDs. OCRA index is computed as the ratio between the number of actual technical actions (TA) performed by the worker during the work cycle and the number of recommended technical actions; a technical action is defined as a combination of manual elementary movements carried out to achieve an operation. The number of recommended technical actions is obtained as the production of several weights that depend on repetitiveness of tasks, i.e., frequency factor, postures, exerted force, work cycle and recovery period, as reported in [2]. Finally, the OCRA index can fall into different five risk areas [7]. When OCRA values fall into the highest risk range, improve working conditions by redesigning tasks and workplaces, activate the health surveillance and enhance the worker training are the main suggested actions to undertake [8].

As reported by several studies [1,5,9], the main disadvantage of this procedure is related to the time consumption required for the computation of all considered factors, especially the counting of the technical action to estimate the frequency factor. For this reason, a simplified version, named as OCRA checklist, has been proposed and validated for a more easy and rapid assessment of working-related MSDs [10]. Conversely to the OCRA index, OCRA checklist requires the counting of the technical actions considering all the workcycle without an analytic partition in several subphases. Following the checklist, score from 0 to 10 is assigned to the frequency factor according to twelve ranges; more specifically, ranges vary from 22.5 actions/minute to greater than 72.4 actions/minute and each range differs from the previous one of 5 actions/minute. The authors proposing the OCRA checklist suggested its use only for an initial screening of the workspace to examine [11]. The computation of OCRA is performed in several industrial settings, such as manufacturing [12], large-scale retail stores [13], assembly [14,15], ergonomics [16], automotive [17] and production processes [18].

In the last decades, many efforts have been made in order to automatize the computation of OCRA index [1,19,20], which means to automatically provide the final value of exerted force, posture and frequency factor. To date, several studies have been proposed for a quantitative evaluation of parameters related to the exerted force and posture. By focusing on perceived force evaluation, Romano et al. proposed to use piezoresistive sensors placed on scissors to quantitatively evaluate the hand force exerted during manual vine branches cutting [21]. The authors verified that the proposed procedure allowed to overcome the demonstrated great variability associated with OCRA index in the identification of forces applied on the scissors. Motion analysis systems have been used for the automatic assessment of postural indices [15,22]; specifically, Savino et al. validated an innovative fuzzy-based system able to quantify the posture by integrating motion analysis data and artificial intelligence routines [23]. Focusing on the frequency parameter needed to evaluate the OCRA index, the counting of technical actions is, indeed, still performed through visual analysis of videos of the workspaces [14,17,18,24]; more specifically, the evaluator firstly recognizes and then counts the technical actions. Actually, the recognition is not required by the OCRA procedure; however, it helps the evaluator in the final counting.

Nevertheless, the video analysis is actually considered the standard procedure; it has been demonstrated that it leads to an incorrect estimation of MSDs risk due to more than 30% of intra-operator variability [25]. In fact, a failed counting of TA affects the final calculation of the OCRA value and, consequently, it implies an erroneous definition of the risk area. Due to this issue, the intra- and inter-rater reliability of the OCRA is still questioned [9,26], even though it is shown among the most reliable procedures for such goal [16]. Spielholz et al. proposed an automatic methodology for counting the repetitions of wrist movements by using an electrogoniometer; however, they obtained a low level of accuracy when comparing data with the ones gathered by the video analysis [27]. Moreover, Lenzi et al. proposed a toolbox for helping evaluators in counting TA; in particular, the designed platform allows the video to run eight time slower and the evaluator can take note of the number of technical actions by pushing button “+” or “−“ [28].

Thus, to the best of the authors’ knowledges, the validation of an automatic procedure for the recognition and counting of the TA needed for the computation of the frequency factor, mandatory for the OCRA value, is still required. From this perspective, this paper extends the preliminary work presented in [29] and aims at understanding if it is possible to find an innovative experimental procedure able to automatically count dynamic technical actions needed for the computation of the frequency factor. To achieve this aim, we proposed a novel procedure and tested it in industrial scenarios, by discussing four different case studies. More specifically, the procedure is based on a threshold-based algorithm capable to compute the frequency factor associated with the OCRA. It is worth highlighting that the procedure only takes into account the dynamic technical action. The outcomes of this study could represent a further step to minimize the incorrect computation of the frequency factor related to the OCRA procedure due to the observational evaluations that are less accurate and operator-dependent.

## 2. Materials and Methods

### 2.1. Industry-Based Protocol: Case Studies

Twenty industrial workers were enrolled in the industrial-based protocol; more specifically four case studies were considered in this study and data related to five participants for each case study was acquired. We selected four different scenarios based on the number of technical actions per minute, addressed as frequency factor, representative of each examined workcycle. In particular; we tested a workcycle with: (i) low frequency, i.e., less than 20 actions per minute; (ii) medium frequency, i.e., actions ranging from 20 to 40 actions per minute; (iii) high frequency, i.e., actions ranging from 40 to 60 actions per minute; and, (iv) higher frequency, i.e., more than 60 actions per minute. The frequency ranges are in line with the ones reported in the OCRA methodology for assigning score to the frequency factor [10]. This choice allowed us to evaluate the goodness of the proposed procedures taking into account different action frequency ranges. In addition, the study on four different industries permits the quantification of the flexibility of the proposed algorithm to different applications.

#### 2.1.1. Low Frequency Scenario

We selected a workspace related to a medium enterprise addressed to the production of nuts and bolts. During the examined work cycle, which lasted approximatively 5 min, the worker performed box-picking operations in the store with the aim of composing a pallet according to the required purchase order. Workcycle is composed also by rest periods in which worker is waiting for the next pallet. Performed technical actions involved both the upper limb sides for the entire workcycle.

#### 2.1.2. Medium Frequency Scenario

We selected a workspace related to an automotive large enterprise. During the examined workcycle, which lasted approximatively 5 min, worker performed assembly operations relatively to the motorbike engine placed on an adjustable-height table. During the examined workcycle, a worker walked within the workspace to take and place different tools. Performed technical actions mainly involved the use of the dominant upper limb that was the right one in the examined case.

#### 2.1.3. High Frequency Scenario

We selected a workspace related to a large industrial laundry. During the examined work cycle, which lasted approximatively 5 min, a worker placed two t-shirts at a time on an ironing surface and actuated the rotary presses to iron the clothes. During the time spent ironing, the worker prepared two other t-shirts on a second ironing surface. When the t-shirts were ironed on both sides, the worker placed them into a cart. The performed technical actions involved both the upper limb sides for the entire workcycle, with a minimal prevalence of the dominant side, which was the right one in all the examined workers.

#### 2.1.4. Higher Frequency Scenario

We selected a workspace related to a medium enterprise for kitchen furniture production. During the examined workcycle, which lasted approximatively 5 min, the worker performed finishing operations for realizing the sideboard doors. Performed technical actions mainly involved the right upper limb.

### 2.2. Data Processing

**Figure 1:**
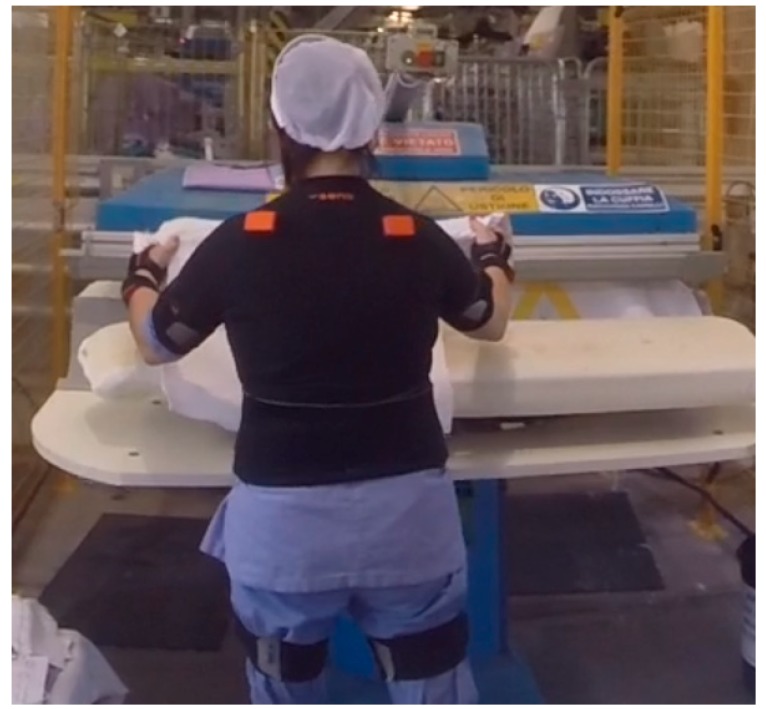
Placement of inertial sensors on a worker. Sensors are the orange probes, when visible, or inside the black belt.

Linear accelerations and angular velocities were gathered from 17 wearable inertial sensors (MVN Biomech Awinda, Xsens Technologies, The Netherlands) positioned on the following body segments: head, neck, 8th and 10th thoracic vertebra, 3rd and 5th lumbar vertebra, right and left shoulder, right and left arm, right and left forearm, right and left hand, pelvis, right and left thigh, right and left shank, right and left foot, and, right and left forefoot. Inertial sensors were selected among other wearable sensors due to their widespread use in applications for human motion recognition [30,31,32,33,34]. Figure 1 shows the placement of inertial sensor on a worker in an industrial scenario.

Inertial sensors attached on the body segments allowed us to acquire linear accelerations, angular velocities and Earth magnetic field with a sampling frequency set at 60 Hz. Angle curves in the three anatomical planes, which are sagittal, frontal and transversal, were successively computed by applying a biomechanical model implemented into the software used for the data analysis (MVN Analyze—Xsens Technologies, The Netherlands). Only angles related to the shoulders, elbow and wrist were considered in this study since OCRA index is focused on the risk assessment of the upper limbs [7]. Thus, the experimental setup can be simplified by only using seven wearable inertial sensors.

A skilled operator identified and counted the technical actions through the analysis of the video recorded during the experimental protocol. Outcomes of video analysis were used as reference to validate the proposed procedure. Details of the procedure are reported in the following subparagraph.

#### Threshold-Based Algorithm for Counting (TB)

**Figure 2:**
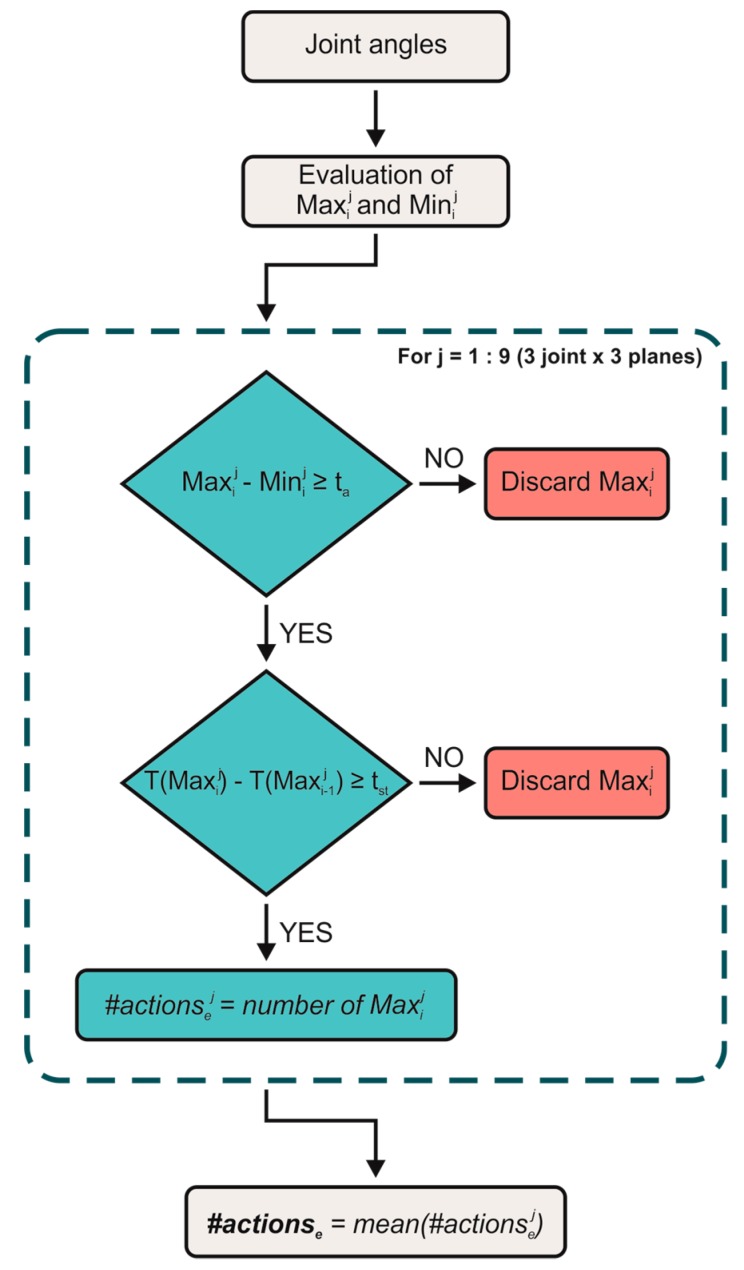
Flowchart of the threshold-based (TB)-procedure.

The threshold-based consists in searching all the maxima and minima of the angle curves related to each of the nine j-*th* angles, which were shoulder, elbow and wrist in the three anatomical planes, applying two thresholds. In Figure 2 is reported the flowchart of the TB-procedure. Specifically, for each angle, the algorithm counts only the maxima (Max_i_) for which (i) the amplitude distance from the successive minimum (Min_i_) was greater than the amplitude threshold (t_a_) and (ii) the time distance, i.e., T(Max_i_)-T(Max_i-1_), from the previous maximum was greater than the time threshold (t_t_). In particular, t_a_ was set to 3°, 5° and 10° allowing to cover both fine motor movements and ones with a greater range of motion and t_t_ was set to 100 ms, 300 ms and 500 ms in order to evaluate different frequencies of the work cycle, coherently with the ranges of OCRA computation. For each j*-th* angle, the number of maxima that passed the two thresholds represents the number of TA ($\#actions_{e}^{j}\;$). The threshold-based algorithm was applied to angles related to each joint (shoulder, elbow and wrist) and each plane (sagittal, frontal and transversal) obtaining 9 numbers of actions for each signal. Finally, the mean value of the number of actions ($\#actions_{e}\;$) was computed and it was compared with the actual number of the technical actions counted by means of the video analysis ($\#actions_{a}$). The procedure was applied by varying in turn each possible couple of t_a_ and t_t_; thus, a total of nine (3 × 3) algorithms were obtained and tested. 

This procedure was applied to the four case studies in industrial scenarios. Frequency Factor (FF) was obtained by dividing for the minutes related to the entire duration of the examined work cycle. Successively, threshold-based algorithm performance in computing FF was assessed as:

$$\varepsilon_{r}\left(\%\right)=\frac{\left|FF_{a}-FF_{e}\right|}{FF_{a}}*100$$

where FF_a_ and FF_e_ represent the actual frequency factor and the estimated one, respectively. Relative error can assume any value from 0%, greater values indicate greater error. Finally, we computed the mean values and standard deviations of $\varepsilon_{r}\left(\%\right)$ by considering the five participants for each case study.

Finally, for each case study, relative error values were firstly tested for normality using the Shapiro-Walking test. Successively, a one-way ANOVA test was used to highlight the presence of statistical differences among the nine tested combinations of the TB algorithm. When the ANOVA test was significative, a Bonferroni’s test for multiple comparisons was applied. All tests were performed by considering a significance level equal to 0.05.

Although it was demonstrated that threshold-based algorithms are less robust in activity recognition rather than machine-learning algorithms [34], it should be underlined that machine-learning algorithms cannot prescind from the training phase and they should be performed for each worker and each industrial workspace, making it difficult to apply these procedures in a real industrial scenario. For this reason, we decided to implement a threshold-based algorithm, also taking into account that this methodology is often used in human motion identification [35,36,37].

## 3. Results

### 3.1. Case Study 1: Low Frequency Scenario

**Figure 3:**
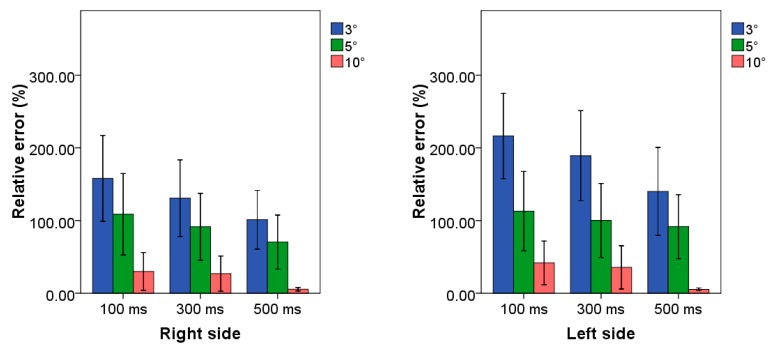
Relative errors related to the case study at low frequency for both side of all combinations of TB procedure. Blue, green and red bars represent the combination in which the threshold amplitude is set to 3°, 5° and 10°, respectively. Clusters on *x*-axis represent the possible time threshold value. Each bar corresponds to a specific combination among the nine tested algorithms.

Mean values and standard deviations of relative errors related to the application of all the examined combinations of TB procedure are reported in Figure 3 for both upper limb sides.

From the analysis of the histograms, we found an average relative error equal to 5.6% and 5.3% as the minimum value achieved for the right and left side respectively associated with the combination 500 ms–10°. Statistical analysis showed statistical differences between such combination and the others. Error values close and higher than 100.0% were obtained for all the combinations in which the amplitude threshold was not set to 10°.

### 3.2. Case Study 2: Medium Frequency Scenario

**Figure 4:**
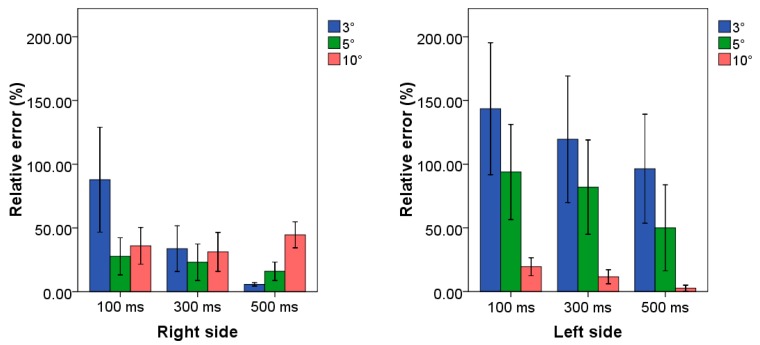
Mean value and standard deviation of relative error related to the case study at medium frequency for both side of all combinations of TB procedure. Blue, green and red bars represent the combination in which the threshold amplitude is set to 3°, 5° and 10°, respectively. Clusters on the *x*-axis represent the possible time threshold value. Each bar corresponds to a specific combination among the nine tested algorithms.

Mean values and standard deviations of relative errors related to the application of all the examined combinations of TB procedure are reported in Figure 4 for both upper limb sides.

By analyzing the results, opposite behaviors can be observed for the two upper limb sides. More specifically, two out nine combinations allowed us to reach average relative error values lower than 17.0%, with a minimum average value of 5.7% regarding the combination 500 ms–3° for the right side. This combination was also found the one with the lowest value of standard deviations. Looking to the left side, the lowest error value was associated with the combination 500 ms–10°.

### 3.3. Case Study 3: High Frequency Scenario

**Figure 5:**
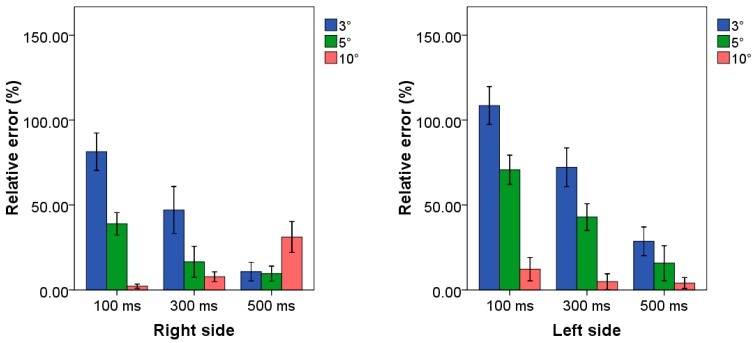
Mean value and standard deviation of relative error related to the case study at high frequency for both side of all combinations of TB procedure. Blue, green and red bars represent the combination in which the threshold amplitude is set to 3°, 5° and 10°, respectively. Clusters on *x*-axis represent the possible time threshold value. Each bar corresponds to a specific combination among the nine tested algorithms.

Mean values and standard deviations of relative errors related to the application of all the examined combinations of TB procedure are reported in Figure 5 for both upper limb sides.

By observing the results, it can be assessed that the lowest value of relative error was associated with the combination 100 ms–10°, i.e., 2.2%, and for the combination 500 ms–10°, i.e., 5.7%, for the right and left side, respectively. Regarding the right side, five out nine combinations allowed us to reach average relative error values lower than 17.0%.

### 3.4. Case Study 4: Higher Frequency Scenario

**Figure 6:**
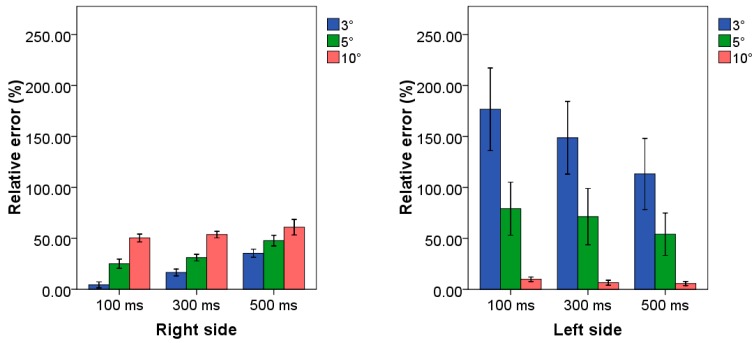
Mean value and standard deviation of relative error related to the case study at higher frequency for both side of all combinations of TB procedure. Blue, green and red bars represent the combination in which the threshold amplitude is set to 3°, 5° and 10°, respectively. Clusters on the *x*-axis represent the possible time threshold value. Each bar corresponds to a specific combination among the nine tested algorithms.

Mean values and standard deviations of relative errors related to the application of all the examined combinations of TB procedure are reported in Figure 6 for both upper limb sides.

By observing the figure, we can affirm that only the combination 100 ms–3° allowed us to reach a reasonable value for the right side; conversely, all the combinations in which the amplitude threshold was set to 10° allowed us to reach a low relative error when looking to the left side. In fact, all the combinations with t_a_ set at 10° allowed us to reach a relative error lower than 10.0%, with the lowest average value associated with the combination of 500 ms–10°, i.e., 5.7%.

## 4. Discussion

### 4.1. Case Study 1: Low Frequency Scenario

By analyzing the results in terms of time threshold, it can be observed as the increment of the threshold value guarantees a constant decrement of the relative error for both upper limb sides. This outcome can be justified by considering the total number of technical actions performed during the examined workcycle; in fact, two technical actions are in general spaced every approximatively three seconds, i.e., 112 actions in five minutes. It emerges that the selection of a time threshold six times lower than the time between each technical action allows achieving optima results; conversely, lower values of time threshold lead to the counting of false positives, which are the identification of technical action not related to the examined workcycle. In addition, a further increase of the time threshold value could cause a misclassification when the frequency increases within the examined workcycle. Concerning the amplitude threshold, a similar trend can be observed; in fact, the increase of t_a_ value permits decreasing the relative error. This suggests that the selection of a low value of amplitude threshold leads to the classification of movements not associated with the technical actions. In summary, the best performance was obtained by using the combination 500 ms and 10° for both upper limb sides. This result is also confirmed by the statistical test that showed that this combination allowed reached statistically lower values of relative error with respect all the other combinations with the exception of 100 ms–10° and 300 ms–10° for both sides. However, the absence of statistical differences can be ascribed to the higher standard deviation associated with the two combinations, which led to a relative error up to 60%. Conversely, the lowest values of standard deviation were associated with the best combination, allowing to affirm its robustness to the inter-subject variability. By analyzing these findings, we can affirm that in this case study, the appropriate selection of the both threshold values appears to be mandatory for the achievement of good/optimum performance. In addition, this application allows affirming that the proposed procedure can work also in presence of internal rest periods during the work cycle.

### 4.2. Case Study 2: Medium Frequency Scenario

Concerning the right side, the results show that the proper selection of a specific time threshold value and amplitude threshold allows us to reduce the relative error by more than 50%. In addition, the best combination was related to a lower value of standard deviation across subjects that permits us to affirm its robustness to the inter-subject variability. The highest values of relative error were generally obtained when the amplitude was set to 10°, with the exception of the combination 100 ms–3° that was associated with the worst performance. The results were also supported by statistical test that showed the presence of statistical differences among the best reported combinations and all the other (*p-value* ranged from <0.01 to 0.04).

Regarding the left side, similar considerations to the previous case study can be carried out for both time and amplitude threshold. This result is in line with the previous examined case study and it can be justified by similar number of total technical actions during the workcycle. This combination was also found to be statistical different from the other with the exception of the other two combination with the amplitude threshold set to 10°. However, we can affirm that the combination 500 ms–10° can be considered the best due to the lowest value of standard deviation, which guarantees robustness across different workers.

These opposite results for the two upper limb sides can be ascribed to the examined work cycle in which the right side was the one mainly involved. Thus, the application of the procedure to this case study allows affirming that the selection of the appropriate threshold values, especially the amplitude one, should derive from the identification of the most involved upper limb and by selecting different values between the most and the least involved side.

### 4.3. Case Study 3: High Frequency Scenario

The best combination was also characterized by the lowest standard deviation, i.e., 1.2%, allowing us to assess its robustness to the inter-subject variability. The results were confirmed by the statistical test, which allowed us to assess that the best combination was statistically different from others (*p-value* ranged from <0.01 to 0.02). By observing the results related to the left side, we can affirm that setting the amplitude threshold value to 10° represents the better choice to achieve the highest performance, regardless of the time threshold value, since average relative errors were always lower than 12.5%. These findings can be related to the types of performed technical actions during the examined work cycle; in fact, no fine movements were required of the worker for completing the technical operations by the non-dominant arm. This implies that the highest value of amplitude threshold avoids counting movements characterized by a small range of motion that is not related to the workcycle. In addition, we can affirm that the two combinations, characterized by a time threshold greater than 100 ms, guarantee both the lowest average relative error values, 4.9% and 4.0% respectively, and a greater robustness to the inter-subject variability due to the limited values of standard deviation. The three combinations characterized by the amplitude threshold set to 10° were also statistically different from other (*p-value* ranged from <0.01 to 0.03). However, we can speculate that the combination 500 ms–10° can be considered as the best one since it allowed us to achieve a maximum relative error equal to 8.0% considering all the examined worker, while the other two were characterized by errors up also greater than 10.0%.

### 4.4. Case Study 4: Higher Frequency Scenario

As regards the right limb, which was the mainly involved in the workcycle, it can be observed as the increment of the time threshold value, from 100 ms to 300 or 500 ms, increases the relative error, especially for the combination with 3° as the amplitude threshold. This outcome can be justified by considering the total number of technical actions performed during the examined workcycle; in fact, two technical actions was in general spaced approximately every 200 ms. It emerges that the selection of a time threshold three times lower than the time between each technical action allows us to achieve the optima results; conversely, higher values of time threshold entail the incorrect counting due to several technical actions that are not correctly identified. The selection of the appropriate amplitude threshold value also appears to be mandatory, since even though the proper time threshold is selected, a relative error greater than 20.0% is obtained if considering 5° or 10° as t_a_. By summarizing, only the combination 100 ms and 3° allowed us to reach an average relative error value of 4.4%. In addition, the low standard deviation permits us to affirm the robustness of the algorithm to the inter-subject variability. This finding is also confirmed by the statistical results that assessed the presence of statistical difference among this combination and others (*p-value* always lower than 0.01). The selection of the appropriate threshold is strictly correlated to the type of the examined work cycle; in fact, worker performed fast and fine movements with both upper limb sides to achieve the technical goal of the work cycle. Thus, it is easy to understand that the lowest values of amplitude and time threshold allow us to correctly count all the fast and fine movements rather than other threshold values. It is worth highlighting that if the evaluator applied the OCRA index, he could be unable to distinguish all the technical actions due to the high frequency of the movements; thus, the evaluator’s results would be less accurate. Conversely, the OCRA checklist would assign the maximum value of FF, i.e., greater than 72 actions/minute, without effectively counted the actions. By using TB procedure, the counting of actions that are not easily distinguishable by the human eyes can be also performed, giving more accurate results.

By considering the left side, which was the one less involved during the working cycle, a proper selection of the amplitude threshold appears to be the only relevant for reaching optimum value of accuracy. The standard deviation values allow stating that the results are consistent across different subjects. Vice versa, the selection of the time threshold appears to be less relevant since similar values were obtained with 100, 300 and 500 ms after the selection of t_a_. These results were also confirmed by the statistical test; in fact, the three combinations were found to be statistically different from the others (*p-values* always lower than 0.01).

### 4.5. General Discussion

To summarize the above-reported results, Table 1 shows the TB combinations that allowed us to reach the best results for all the four examined case studies.

By analyzing the table, it is clear that a best-performing combination equal for all the examined case studies and for both upper limb sides is not identifiable. This finding allows us to state that the TB procedure is work-dependent; however, a combination that allows reaching an average relative error lower than 6.0% is obtainable in all the examined workcycles after the proper selection of t_t_ and t_a_. As depicted in the table, t_t_ equal to 300 ms and t_a_ equal to 5° are selected in no one of the best combinations; thus, we can affirm that the operator should select only between two values of t_t_ and t_a_ to achieve the optima results. In addition, we would like to underline that the application of TB procedure guarantees in all examined cases the selection of the same frequency factor score if we consider the OCRA checklist. In fact, in all four examined cases and for both upper limb sides the estimated number of TA and the average relative error did not cause a different selection of frequency factor range and, consequently, the same score of the OCRA checklist can be assigned. Indeed, if the relative error of 7% caused an incorrect assignment of the frequency factor score, it would be always less than the error rate demonstrated to be associated with the observational methods [25]. Thus, we can affirm that the found relative error should be considered negligible for OCRA computation, especially if the checklist is applied.

Considering the results, we can state that the appropriate selection of amplitude and time threshold base cannot be bypassed. Thus, the operator addressed to TA counting for the definition of the OCRA frequency factor should get preliminary information related to the workcycle. Specifically, the operator should insert into the algorithm: (i) a qualitative information about the gesture performed during the work cycle, i.e., fine or not; (ii) if both upper limb sides are involved in the execution of technical actions; (iii) a qualitative information about the speed of gesture, i.e., slow or fast movement. As a solution, the operators should perform a short initial pre-counting through the reported guidelines and, then, select the appropriate threshold values for the final counting. Furthermore, after the proper selection of the two threshold values, they can be used for all workers and for all workcycles similar to the already tested one, avoiding the pre-counting phase. Alternatively, if a greater accuracy is required, the operator could apply all the combinations of the threshold-based algorithms to a workcycle for which he/she knows the actual number of the technical actions in order to obtain the most appropriate values of t_t_ and t_a_ and, then, to use them for the successive applications on the same workcycle. In fact, the found relative error is significantly lower than 30%, which has been demonstrated to be the possible error related to the observational procedure [25]. In addition, the found standard deviations associated with all the best combinations (maximum value 3.2%) guarantee to reach a maximum relative error lower than 10.0%. It is worth highlighting that the combinations associated with the lower average relative error is also related to the lower standard deviation. This finding allows us to state that the TB procedure works well also with different subjects if the correct combination is selected.

The TB procedure is not useful only as a tool for OCRA checklist computation but also for the more complex computation of the OCRA index frequency factor if the appropriate thresholds are selected. For these reasons, the TB procedure reveals itself as a useful tool to bring to the calculation of the technical actions, helping the operator to make a more objective analysis. In addition, such an approach could allow us to reduce the intra- and inter-subject variability related to the OCRA, or more generally to all the observational methodologies, such as Strain Index and ACGIH (TLV) [26]. A further advantage related to the TB procedure is that it could be applied also in complex tasks, i.e., multiple task jobs, such as the ones in case study 2. Conversely, the FF computation via observational methods is considered onerous in these types of work [24].

It is also evident as the TB procedure could allow us to drastically reduce the time-consumption in OCRA frequency factor computation; more specifically, just to report a quantitative value, the time spent in the computation of FF on an entire work cycle, with a duration of 5 min, is about 150 ms. This reduction will be especially emphasized in a long workcycle. Nowadays, the FF video analysis of the same workcycle approximatively requires from 10 to 20 min. Actually, the time efforts required for the evaluation of OCRA value via observational methods leads to the operator tiredness that can cause an inaccurate computation of OCRA to all the workcycles for each work-phase of the production chain. In fact, the operator generally computes the OCRA only on a few workcycles for each work-phase and he/she assumes that all other cycles are similar to the examined one [1]. Conversely, the computation of the OCRA index is often so heavy that operators do not compute it. Thus, the introduction of TB procedure as an evaluation tool could allow us to enlarge the number of examined workcycles for each worker and the number of the examined workers due to the drastic reduction of the time-consumption, achieving a more robust and objective risk assessment.

Furthermore, TB application can guarantee the possibility to perform the action counting only for some body segments whether operator aims at identifying specific pathologies, such as carpal tunnel syndrome and epicondylitis, since some authors suggested the evaluation of only the involved body segments, such as the wrist and the shoulder [8]. Finally, TB can be used to evaluate the number of technical actions related to specific anatomical planes that, conversely, are out of the question in video analysis due to the 2D nature of the data [38].

Although this study provides a preliminary contribution for the automatization of the OCRA computation, further studies have to be carried out focused on the optimization of the threshold value selection by evaluating the reliability of the proposed procedure.

## 5. Conclusions

Through the aim of proposing an automatic procedure for the computation of the dynamic frequency factor related to the OCRA methodology to reduce observational analysis effort, we proposed an innovative threshold-based algorithm. The procedure was tested in four industrial scenarios, concerning low, medium, high and higher frequency scenario in terms of rapidity to execute technical actions within the workcycle. The algorithm was based on two threshold values, related respectively to the time and the amplitude of the action identified in joint angle curves of the upper limbs. By analyzing the results, we can affirm that a proper selection of time and amplitude threshold values represents a mandatory step for obtaining optimum performance. In particular, average relative errors lower than 6.0%, with the threshold-based procedure proposed here were obtained after threshold selection. This value can be considered acceptable, especially considering the higher error and inter-operator variability related to the video analysis approach. In conclusion, the innovative approach allows us to both drastically reduce the time spent in frequency factor identification by video analysis and avoid the modest inter-operator reliability of observational methods. Future works will be finalized to reduce the number of sensors by identifying the most involved joint in the specific workcycle and to assess the intra-worker reliability of the procedure.

## Figures and Tables

**Table 1:** The best-performing combination for all the examined industrial case studies with related mean value and standard deviation of the relative error. TB combination stands for the time (ms) and the amplitude (°) threshold value set for reaching the lowest relative error.

		Low Frequency	Medium Frequency	High Frequency	Higher Frequency
**Right Limb**	**TB Combination**	**500 ms–10°**	**500 ms–3°**	**100 ms–10°**	**100 ms–3°**
	*Relative Error (%)*	5.6 (2.5)	5.7 (1.3)	2.2 (1.2)	4.4 (2.9)
**Left Limb**	**TB Combination**	**500 ms–10°**	**500 ms–10°**	**500 ms–10°**	**500 ms–10°**
	*Relative Error (%)*	5.3 (1.2)	2.6 (2.4)	4.0 (3.2)	5.7 (2.1)
